# Computed Tomography Assessment of the Bronchial Lumen–Vertebral Body and Pulmonary Artery–Vertebral Body Relationships in Cats Naturally Infected with Immature *Dirofilaria immitis*

**DOI:** 10.3390/vetsci13020186

**Published:** 2026-02-13

**Authors:** Sara Nieves García-Rodríguez, Jorge Isidoro Matos, Laín García-Guasch, Eva Mohr-Peraza, José Alberto Montoya-Alonso, Elena Carretón

**Affiliations:** 1 Internal Medicine, Faculty of Veterinary Medicine, Research Institute of Biomedical and Health Sciences (IUIBS), University of Las Palmas de Gran Canaria, 35016 Las Palmas de Gran Canaria, Spain; saranieves.garcia@ulpgc.es (S.N.G.-R.); lain.garcia@ivcevidensia.es (L.G.-G.); eva.mohr@ulpgc.es (E.M.-P.); alberto.montoya@ulpgc.es (J.A.M.-A.); elena.carreton@ulpgc.es (E.C.); 2 IVC Evidensia Hospital Veterinari Molins, 08005 Barcelona, Spain; 3 IVC Evidensia Hospital Veterinaria del Mar, 08005 Barcelona, Spain

**Keywords:** bronchial remodelling, CT, dirofilariosis, feline, HARD, heartworm, thorax

## Abstract

Feline heartworm disease, caused by *Dirofilaria immitis*, is often underdiagnosed, particularly during its larval stage, known as Heartworm-Associated Respiratory Disease, (HARD). This study aimed to quantify CT-detectable pulmonary changes in naturally infected cats. Computed tomography (CT) was performed in 38 cats: Group A (n = 30, symptomatic seropositive) and Group B (n = 8, asymptomatic seronegative). Bronchial and pulmonary artery diameters were indexed to the sixth thoracic vertebral body (T6) to calculate bronchial-to-vertebral body (B/VB) and pulmonary artery-to-vertebral body (A/VB) ratios across all lung lobes. Group A cats showed significantly higher B/VB ratios in the left cranial and right middle lobes compared with Group B (*p* < 0.05), with increases of 42.0% and 47.5%, respectively. Conversely, A/VB ratios did not differ significantly between groups (*p* > 0.05), indicating the absence of relevant vascular remodelling. Intra-operator and inter-observer reliability were excellent (intraclass correlation coefficients > 0.85). These findings suggest that bronchial dilation in the absence of arterial enlargement represents a key tomographic feature of larval *D. immitis* infection. Quantitative CT ratios, particularly the B/VB ratio, provide objective imaging markers for the diagnosis of HARD in clinical practice, and may assist in differentiating this condition from other feline respiratory diseases in endemic areas.

## Simple Summary

Feline heartworm disease, caused by the parasite *Dirofilaria immitis*, is a serious condition that often goes undetected in cats. Unlike dogs, which typically develop infections with adult worms in the heart, cats more frequently experience a larval form of the disease known as Heartworm-Associated Respiratory Disease (HARD), which primarily affects the lungs. Because affected cats often show vague signs such as coughing or breathing difficulty, diagnosing can be challenging. In this study, we used advanced imaging with computed tomography (CT) to examine the lungs of naturally infected cats. By comparing CT measurements from infected cats with those from healthy controls, we found that infected cats showed clear widening of the airways (bronchi), even when the pulmonary arteries appeared normal. To ensure reliable comparisons, lung measurements were standardized using the cat’s spine as a stable reference point. These findings show that specific CT-based lung measurements can help veterinarians detect early-stage heartworm infection in cats more accurately. This approach may improve diagnosis in areas where the parasite is common and support earlier treatment and prevention, ultimately benefiting feline health and welfare.

## 1. Introduction

*Dirofilaria immitis* is a parasitic nematode with a profound clinical impact on the bronchial structures and pulmonary arteries of cats [1,2,3]. In felines, the disease spectrum ranges from Heartworm-Associated Respiratory Disease (HARD), caused by immature larvae, to chronic cardiopulmonary dirofilariosis, resulting from adult parasite infection [3,4]. Despite its clinical relevance, feline heartworm disease remains frequently underdiagnosed. Cats are often perceived as more resistant to *D. immitis* infection than dogs, which contributes to a lower index of clinical suspicion [5,6].

Definitive diagnosis is further complicated by the nonspecific nature of clinical, radiographic, and histopathological findings, which frequently overlap with those of other feline respiratory diseases, including asthma, chronic bronchitis, and parasitic infections such as *Aelurostrongylus abstrusus* and *Toxocara cati* [1,7,8,9]. Consequently, achieving a confirmatory diagnosis typically requires a multimodal approach that integrates laboratory testing with advanced imaging techniques [10,11,12].

Computed tomography (CT) has emerged as a valuable diagnostic tool in this context, providing high-resolution visualization of the vascular remodelling and parenchymal changes associated with *D. immitis* infection [2,13]. In cats with naturally acquired adult heartworm infection, CT has been shown to identify right atrial and ventricular dilation, as well as increased luminal diameter and tortuosity of the pulmonary arteries—findings consistent with pulmonary thromboembolism [14,15]. Beyond vascular lesions, CT can also reveal parenchymal alterations indicative of a restrictive pulmonary pattern, characterized by changes in lung radiodensity and reduced lung volume without evidence of hyperinflation [2]. Experimental studies have further demonstrated that CT is effectively in characterizing the involvement of both pulmonary arteries and bronchial structures [2].

To objectively quantify these alterations, recent experimental studies have employed CT-derived morphometric ratios, including measurements of the bronchial lumen adjacent pulmonary artery (PA) relative to the vertebral body of the sixth thoracic vertebra (T6). These measurements yield the bronchus-to-pulmonary artery (BA), bronchial lumen-to-vertebral body (B/VB), and pulmonary artery-to-vertebral body (A/VB) ratios, in cats experimentally infected with adult *D. immitis* [16]. As T6 is a fixed and stable anatomical landmark, these ratios provide reliable and objective indices for assessing parasite-induced cardiopulmonary changes [17].

While recent CT-based studies in cats seropositive for *D. immitis* and clinically compatible with HARD have reported significant bronchial dilation relative to pulmonary arteries [18], specific evaluation of B/VB and A/VB ratios has not been performed in cats naturally infected with immature stages of *D. immitis*. Characterizing these ratios is essential to better differentiate larval-induced structural changes from those associated with other lower respiratory tract diseases.

Therefore, the aim of the present study was to quantify and compare the B/VB and A/VB ratios in symptomatic seropositive cats and asymptomatic seronegative cats using CT imaging. By doing so, the authors sought to characterize anatomical changes associated with larval *D. immitis* infection and to explore the potential diagnostic utility of vertebral-based morphometric ratios in the clinical setting.

## 2. Materials and Methods

### 2.1. Animal Selection and Study Design

This prospective observational study was conducted between May 2022 and May 2024. A total of 38 cats were enrolled and divided into two groups: Group A (n = 30), comprising cats with suspected Heartworm-Associated Respiratory Disease (HARD) based on the presence of respiratory clinical signs (e.g., coughing, dyspnea, tachypnea) and positive *D. immitis* serology; and Group B (n = 8), consisting of asymptomatic, seronegative control cats. Group B animals underwent CT scanning for unrelated clinical reasons (e.g., trauma or neurological disorders) that did not involve the thoracic structures.

For all animals, a complete clinical record was maintained, which included information on age, sex, breed, body weight, and relevant medical history. The inclusion criteria required cats to be older than six months of age. Additional criteria stipulated that cats in Group A had not received prior heartworm prophylaxis and that no animal had been administered pharmacological treatment before blood sample collection. Inclusion criteria for Group B included the absence of cardiorespiratory clinical signs and a normal physical examination, including normal cardiac and pulmonary auscultation. All included cats tested negative for bronchopulmonary parasitic infections and showed no evidence of cardiovascular or systemic disease that could potentially affect the pulmonary vasculature; therefore, animals with known or suspected cardiac pathology were excluded. Both groups were balanced for age, sex, and body weight to minimize potential confounding effects.

Prior to CT examination, all cats underwent a complete physical examination, thoracic radiography (right lateral and ventrodorsal views) and hematological and renal function assessments to evaluate general health status, particularly in the control group. Written informed owner consent was obtained for all participants, and the study was conducted in accordance with European legislation on animal protection and research ethics. The control group was used exclusively for comparative purposes and was not intended to establish reference values for the feline species.

### 2.2. Serological Analysis

Blood samples were collected via jugular, cephalic, or femoral venipuncture. Serum was isolated by centrifugation and used for the detection of *D. immitis* antigens and antibodies. Circulating antigens were detected using a commercial immunochromatographic test (Uranotest Dirofilaria©, UranoVet SL, Barcelona, Spain), following manufacturer’s instructions. Antibody status was determined via an indirect ELISA (in-house ELISA, UranoVet SL, Barcelona, Spain) utilizing recombinant *D. immitis* Di33 protein as previously described [18]. Briefly, serum samples were diluted 1:100 in sample diluent buffer and added to ELISA plate wells pre-coated with recombinant *D. immitis* Di33 protein (0.5 µg/mL). After a first wash to remove unbound components, a conjugate solution was added to bind antigen–antibody complexes. A second wash was then performed before adding the substrate (TMB) in a dark environment to allow specific binding to feline IgG. The reaction was stopped with sulfuric acid, and optical density (OD) was measured at 450 nm using a spectrophotometer.

According to the manufacturer’s instructions, samples with a cut-off value ≥ 1 were considered seropositive for *D. immitis* antibodies, whereas samples with a diagnostic value < 1 were classified as seronegative.

### 2.3. CT Acquisition and Image Analysis

All seropositive animals underwent CT examination to identify imaging findings compatible with immature *D. immitis* infection. Seronegative animals were imaged for diagnostic purposes unrelated to cardiorespiratory disease (e.g., trauma or neurological disorders).

CT images were acquired using a helical CT scanner (Canon Toshiba Astelion, Canon Medical Systems, Tokyo, Japan). Cats were positioned in sternal recumbency with the head extended cranially. Scans were obtained before and after intravenous administration of a non-iodinated contrast agent (Xenetix^®^, Guerbet, Roissy, France) at a dose of 600 mg/kg. Images were reconstructed using 1 mm slice thickness (pitch factor: 0.94), with algorithms optimized for soft tissue and bone/lung evaluation.

All cats followed a standardized anesthetic protocol. Premedication consisted of intravenous midazolam (0.2 mg/kg; Midazolam, B. Braun Medical, Barcelona, Spain) and butorphanol (0.2 mg/kg; Torphadine^®^, Dechra, Northwich, UK). Anesthesia was induced with propofol (0.6 mg/kg; Propofol Lipuro^®^, B. Braun VetCare, Barcelona, Spain), and maintained with 2.5% sevoflurane (SevoFlo^®^, Zoetis, Louvain-la-Neuve, Belgium) delivered via endotracheal intubation. Cats were maintained under general anesthesia with spontaneous breathing, without the use of mechanical or positive-pressure ventilation. Vital parameters were continuously monitored throughout the procedure.

CT image analysis was performed following previously described protocols [16,17,18]. Vertebral measurements were obtained using the bone window (window width [WW]: 2500; window level [WL]: 480), while bronchial and pulmonary arterial luminal measurements were obtained using the lung window (WW: 1400; WL: −500).

Measurements were performed on transverse (axial) images at predefined anatomical levels. Bronchi and pulmonary arteries were evaluated at the level of the cranial lung lobes (left and right cranial lobes) between T4 and T5; at the level of the middle lung lobe and caudal portion of the left cranial lobe between T6 and T7; and at the level of the caudal lung lobes, including the accessory lobe, left caudal lobe, and right caudal lobe, between T9 and T10.

**Figure 1:**
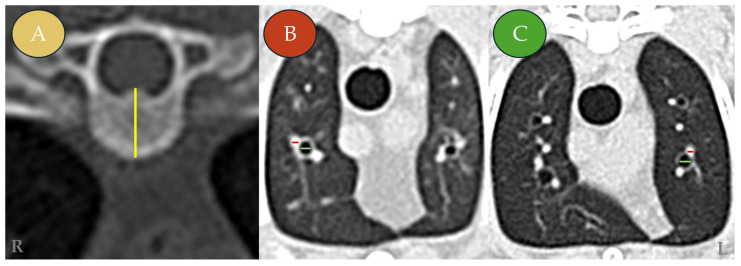
Representative transverse (axial) computed tomography (CT) images of symptomatic *Dirofilaria immitis*-seropositive cats (Group A). The images illustrate the morphometric measurement protocol for the vertebral body, pulmonary arteries, and bronchi. (**A**) Transverse (axial) section, showing measurement of the sixth thoracic vertebral body (T6) height (yellow line) performed along the long axis of the vertebral body, from the dorsal margin of the vertebral canal to the ventral cortical surface, and used for normalization. (**B**) Transverse section at the level of the T4–T5 vertebrae showing the right cranial lung lobe. (**C**) Transverse section at the level of T4–T5 showing the left cranial lung lobe (cranial subsegment). Measurement indicators: pulmonary artery diameter (red line) and bronchial luminal diameter (green line). L: left; R: right.

Bronchial and vascular luminal diameters were measured on axial images in which the structures appeared round or near-round, indicating a perpendicular orientation to the imaging plane. Minor cranial or caudal adjustments were allowed to ensure true transverse orientation, following previously described protocols [16,17]. The height of the sixth thoracic vertebral body (T6) was measured on axial images along its long axis, from the dorsal margin of the vertebral canal to the ventral cortical surface (Figure 1).

All measurements were obtained using electronic callipers placed at the point of maximum luminal diameter. Image stacks were manually scrolled and minimally rotated when necessary to optimize visualization of bronchovascular structures, and image magnification was used to enhance measurement accuracy. All image analyses were performed using Horos software (Version 3, LGPL-3.0). B/VB and A/VB ratios were calculated for each lung lobe according to standardized criteria.

### 2.4. Statistical Analysis

For categorical variables, results were expressed as frequencies and percentages. Differences between groups were assessed using Pearson’s Chi-squared test for non-parametric data, while Fisher’s exact test was applied when the analysis involved 2 × 2 contingency tables.

Normality of continuous data was assessed using the Shapiro–Wilk test. As most continuous data did not follow a normal distribution, between-group comparisons were performed using the non-parametric Mann–Whitney U test. Differences in thoracic vertebra (T6) between groups were assessed using an independent samples *t*-test with Welch’s correction.

B/VB and A/VB ratios were analyzed using mixed linear models (MLMs) for repeated measures. These models accounted for repeated measurements across lung lobes within the same subject and were adjusted for body weight and age. Robust covariance estimators were applied to address potential violations of model assumptions. The statistical analysis was designed to detect differences both between groups (Group A vs. Group B) and among lung lobes.

Specifically, the models evaluated whether significant differences existed in mean ratio values between groups for each lung lobe and whether, within each group, mean ratio values differed among lung lobes. When significant effects were detected, post hoc pairwise comparisons were performed with Bonferroni correction to control for type I error.

Effect size estimates were reported to facilitate interpretation of the results. For continuous variables, Cohen’s d was calculated and interpreted as small (0.2–0.4), medium (0.5–0.8), or large (>0.8). For categorical variables, Cramér’s V was calculated and interpreted as negligible (0.00–0.09), weak (0.10–0.29), moderate (0.30–0.49), or strong (>0.50).

Intra-operator repeatability was assessed using repeated measurements performed by the same operator, while interobserver reliability was evaluated using independent measurements obtained by a second operator. Both operators were veterinarians experienced in diagnostic imaging and CT analysis and were blinded to group allocation.

Intra-operator repeatability was assessed in ten randomly selected cats, each measured on five consecutive occasions in the left and right caudal lung lobes. For each variable, the within-subject standard deviation (SD) was calculated and averaged to estimate intra-operator measurement error. Measurement precision was expressed as the coefficient of variation (CV), calculated as CV = (SD_intra/global mean) × 100. CV values ≤ 5% were considered excellent, and values between 5 and 10% were considered acceptable.

Interobserver reliability was assessed using measurements independently obtained by two observers (S.N.G.-R. and J.I.M.) in the same ten cats and (left and right caudal) lung lobes. Agreement was quantified using the intraclass correlation coefficient (ICC, two-way random-effects model, single measurement, absolute agreement [ICC(2,1)]). Measurement error was expressed as the standard error of measurement (SEM) and coefficient of variation (CV = SEM/global mean × 100). ICC values ≥ 0.75 were considered indicative of good reliability and values ≥ 0.90 of excellent reliability, while CV values < 10% were considered acceptable and <6% indicated of very good precision [19,20].

Statistical significance was primarily set at *p* < 0.05; however, results were also evaluated at α levels of 0.01 and 0.10 to explore trends. No formal sample size calculation was performed, as this was an exploratory study. All statistical analyses were performed using SPSS software (version 25.0; IBM Corp., Armonk, NY, USA).

## 3. Results

### 3.1. Descriptive Analysis

The study included 38 cats: 16 males (42.1%) and 22 females (57.9%). The mean age of 4.32 years (range 1–15) and the mean body weight was 3.56 kg. European Shorthair was the predominant breed (n = 33; 86.8%). No statistically significant differences were observed between Group A and Group B regarding sex (*p* = 0.767), breed (*p* = 0.217), age (*p* = 0.407), or body weight (*p* = 0.208), confirming baseline comparability between groups.

Mean T6 vertebral body height was consistent across the study population, with no significant differences between seropositive cats (5.06 ± 0.54) and seronegative cats (5.07 ± 0.34; *p* = 0.932). Table 1 and Table 2 summarize the absolute diameters of the pulmonary arteries (PA) and bronchial lumens, respectively, across all lung lobes. Descriptive analysis showed that Group A exhibited larger mean bronchial diameters in the left cranial, middle, and left caudal lung lobes compared with Group B. In contrast, mean PA diameters in the right cranial and caudal lobes were slightly larger in Group B.

### 3.2. Pulmonary Artery–Vertebral Body (A/VB) Ratio

**Figure 2:**
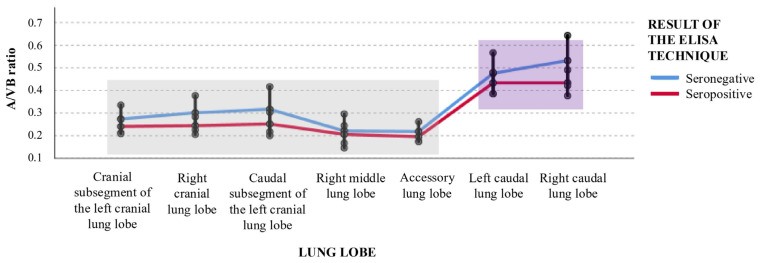
Distribution of the pulmonary artery-to-vertebral body (A/VB) ratio across lung lobes in *Dirofilaria immitis*-seropositive (Group A) and seronegative (Group B) cats. The graph illustrates the consistency of vascular morphometry between both groups across all lung segments (*p* > 0.05). Note the preserved physiological pattern in both groups, characterized by significantly higher A/VB ratios in the caudal lobes (purple box) compared to the cranial, middle, and accessory lobes (grey box) (*p* < 0.05). This parallel distribution supports that larval infection does not induce significant macro-vascular remodelling detectable by CT-indexed ratios.

The mean A/VB ratio did not differ significantly between Group A and Group B in any lung lobe (all *p* > 0.05) (Table 3). However, significant intra-group variations were observed among lung lobes. In both groups, A/VB ratios were significantly lower in the left cranial lobe (both cranial and caudal subsegments), right cranial lobe, right middle lobe, and accessory lobe compared with the caudal lung lobes. These differences were associated with large effect size (Cohen’s d: 1.0–2.0). Accordingly, the left and right caudal lobes consistently exhibited the highest A/VB ratios across the pulmonary parenchyma (Table 3 and Table 4; Figure 2). Within Group B, the accessory lobe showed a marginally lower A/VB ratio compared with the left cranial (cranial and caudal subsegments) and right cranial lobes. This difference was associated with a small effect size (Cohen’s d: 0.3–0.4).

### 3.3. Bronchial Lumen–Vertebral Body (B/VB) Ratio

Statistically significant differences in the mean B/VB ratios were identified between Group A and Group B in the left cranial lobe (cranial subsegment) and the right middle lobe (*p* < 0.05). In both regions, cats in Group A exhibited markedly higher B/VB ratios. Specifically, mean B/VB ratios were 42% higher in the left cranial lobe and 47.5% higher in the right middle lobe compared with Group B, corresponding to a medium effect size (Cohen’s d ≈ 0.5).

A trend toward increased B/VB ratios in Group A was also observed in the right cranial lobe and in both caudal lobes; however, these differences did not reach conventional statistical significance (*p* < 0.1) (Table 5).

**Figure 3:**
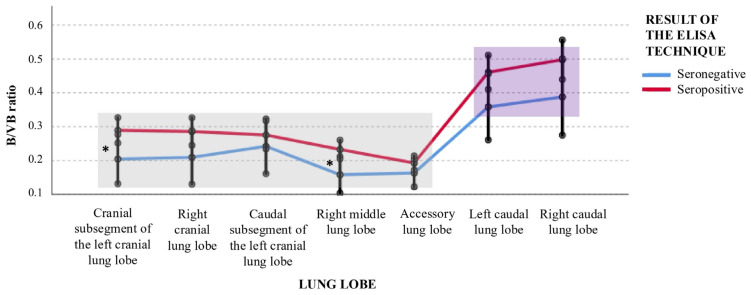
Distribution of the bronchial lumen-to-vertebral body (B/VB) ratio across lung lobes in *Dirofilaria immitis*-seropositive (Group A) and seronegative (Group B) cats. Two distinct anatomical clusters are identified: the caudal lobes (purple box), characterized by higher and more uniform ratios, and the remaining lung lobes (grey box). Asterisks (*) denote statistically significant differences (*p* < 0.05) between Group A and Group B within specific lobes, indicating bronchial dilation associated with Heartworm-Associated Respiratory Disease (HARD). The overall distribution demonstrates that while caudal lobes maintain higher baseline dimensions, the inflammatory response to larval infection significantly alters bronchial morphometry in the cranial and middle lung segments.

Intra-group analysis revealed a lobar distribution pattern similar to that observed for vascular parameters. B/VB ratios were significantly lower in the left and right cranial lobes, right middle lobe, and accessory lobe compared with the left and right caudal lobes, which showed higher and more homogeneous values. These differences were associated with large effect sizes (Cohen’s d: 1.0–2.0; Table 5 and Table 6; Figure 3).

Overall, Group A showed increased bronchial dimensions in specific lung lobes, whereas vascular dimensions remained largely unchanged between groups. Within-group analysis revealed that caudal lung lobes consistently exhibited higher bronchial and vascular ratios than cranial and middle lobes.

### 3.4. Measurement Precision and Reliability

Intra-operator repeatability was high across all evaluated variables, with coefficients of variation (CV) ranging from 4.36% to 6.40% (Table 7). The highest measurement precision for both A/VB and B/VB ratios was observed in the right caudal lung lobe.

Inter-observer reliability was also excellent, with intraclass correlation coefficients (ICC) ranging from 0.859 to 0.978 (Table 8). The A/VB ratio measured in the right caudal lobe showed the highest level of agreement between observers (ICC = 0.978).

Absolute measurement error remained low, with standard error of measurement (SEM) values ranging from 0.0156 to 0.0266. Relative measurement error was also low, with CVs between 4.39% and 5.28%, all of which fell within acceptable clinical limits (CV < 10%) [19,20].

## 4. Discussion

CT has become an indispensable diagnostic tool in feline medicine, particularly for thoracic evaluation [15,21]. Its ability to generate high-resolution, cross-sectional images allows detailed assessment of pulmonary, bronchial, and vascular structures, surpassing conventional radiography in both sensitivity and specificity [22,23]. CT has demonstrated diagnostic utility in a wide range of pulmonary conditions, including bronchiectasis, asthma, chronic bronchitis, and parasitic diseases such as those caused by *D. immitis* [16,24,25,26,27,28,29]. In this context, the present study exploits these advantages to provide a quantitative imaging framework for the diagnosis of feline heartworm infection, specifically its larval manifestation, Heartworm-Associated Respiratory Disease (HARD).

A major challenge in feline dirofilariosis is its nonspecific clinical presentation and the frequent absence of adult parasites, which makes HARD a perennially underdiagnosed condition. While most previous research has relied on experimental infections [2,16], the present study is among the first to establish normalized morphometric indices—bronchial and arterial diameters relative to the T6 vertebral body (B/VB and A/VB)—in a naturally infected, symptomatic feline population [18].

The most relevant finding of this study was the significant elevation of B/VB ratios observed in symptomatic seropositive cats, particularly in the left cranial and right middle lung lobes. This pattern is consistent with the known pathophysiology of HARD, in which the arrival of immature *D. immitis* larvae in the pulmonary vasculature triggers an intense eosinophilic and lymphocytic inflammatory response, leading to airway remodelling and bronchial dilation [2,11]. These lobes are also recognized as being predisposed to collapse or consolidation in feline asthma, particularly the right middle lobe, owing to its dorsoventral orientation and susceptibility to mucus accumulation, which can result in atelectasis and mediastinal shift. However, no consistent CT findings of atelectasis, mediastinal shift, or mucus accumulation were identified in the cats included in this study, indicating that the observed bronchial enlargement was not associated with positional or obstructive parenchymal changes. Similar bronchial involvement has also been reported in the caudal subsegment of the left cranial lobe [30]. As these imaging patterns may overlap with those seen in feline asthma, neoplasia, or chronic bronchitis [31,32], the application of objective and quantitative CT ratios represents a valuable tool for improving differential diagnosis.

The dispersion of bronchovascular ratios observed across lung lobes is likely attributable to inherent anatomical and physiological variability rather than to measurement inconsistency. Bronchial and vascular structures in cats vary according to lobar anatomy, branching pattern, and functional demand [33], which can result in broader value ranges when measurements are obtained across multiple pulmonary regions. Importantly, similar lobar-dependent dispersion patterns were observed in both seropositive and seronegative cats, supporting a biological rather than methodological origin for this variability. Accordingly, the present results are interpreted in terms of relative lobar differences and group-level patterns rather than absolute diagnostic thresholds.

Regarding vascular measurements, the A/VB ratios in the control group (Group B) were consistently higher in the caudal lung lobes, in agreement with established physiological patterns of feline pulmonary perfusion [17]. Notably, seropositive cats (Group A) did not exhibit significant differences in A/VB ratios compared with controls. These findings contrast with the results reported by Lee-Fowler et al. [16], who described marked pulmonary artery (PA) enlargement in cats experimentally infected with adult heartworms. The absence of vascular remodelling in the present cohort strongly suggests that the pathological process was restricted to the larval stage of infection. Consequently, bronchial dilation in the absence of concomitant arterial enlargement may represent a radiological hallmark of HARD, allowing differentiation from chronic feline dirofilariosis, in which adult-associated vascular changes such as thromboembolism and arterial tortuosity predominate [14,15].

Although Lee-Fowler et al. [16] reported bronchial dilation predominantly affecting the middle and left cranial lobes, similar to the present findings, bronchial changes in this study extended to the caudal lung lobes. This difference may reflect gravity-dependent distribution of larval-induced inflammation in naturally infected cats, or variations in host immune responses in non-experimental conditions.

The lobe-dependent distribution of both B/VB and A/VB ratios warrants further consideration. Caudal lung lobes exhibited higher mean ratios in both groups, consistent with the physiological predominance of perfusion and ventilation in these regions. This anatomical characteristic may increase susceptibility to parasitic inflammatory injury. Interestingly, seropositive cats showed a disproportionate increase in bronchial diameter in caudal lobes, suggesting that larval-induced inflammation may preferentially affect highly perfused areas, potentially through immune complex deposition or localized hypersensitivity reactions.

The use of the T6 vertebral body as a standardized anatomical reference represents a methodological strength of this study. In contrast to previous reports that described bronchial changes without normalization [18], the use of B/VB and A/VB ratios improves reproducibility and enables inter-individual comparisons regardless of body size. This is supported by the absence of significant differences in T6 vertebral body height between groups. This approach provides a foundation for the future establishment of clinically applicable diagnostic thresholds.

Several limitations of the study should be acknowledged. The precise timing of infection could not be determined, as antibody positivity reflects exposure rather than active larval migration. Given the limited number of control cats, the study should be considered exploratory, and the findings interpreted as hypothesis-generating rather than definitive. Additionally, bronchial diameter varies with the respiratory cycle, and the absence of respiratory gating or mechanical ventilation during CT acquisition represents a methodological limitation. Therefore, the measurements should be interpreted comparatively rather than as absolute values. Although feline asthma was included in the differential diagnosis, it could not be conclusively excluded in all cases, and the presence of concurrent or subclinical asthma in some seropositive cats cannot be ruled out. A complete echocardiographic evaluation was not performed to exclude subclinical cardiac disease; however, this is considered unlikely given the absence of clinical and radiological findings suggestive of cardiac pathology. Finally, the relatively small size and unmatched age distribution of the control group may have obscured subtle age-related anatomical differences [17]. Future studies should therefore include larger, age-matched populations, prospective infection monitoring, and respiratory-gated CT acquisition to further refine bronchial measurements.

Overall, these findings reinforce CT as a sensitive, non-invasive diagnostic modality for feline dirofilariosis. In endemic regions, an increased B/VB ratio—especially when associated with a normal A/VB ratio—should raise strong suspicions of HARD, prompting serological confirmation and the timely implementation of appropriate preventive and therapeutic strategies.

## 5. Conclusions

This study demonstrates that natural infection with immature *Dirofilaria immitis* in cats primarily induces bronchial alterations, rather than vascular remodelling typically associated with adult infections. CT-derived morphometric analysis, specifically the B/VB ratio, proved to be a robust and sensitive indicator of airway involvement in HARD. These normalized parameters provide clinicians with a valuable diagnostic tool, particularly in endemic areas where feline dirofilariosis remains frequently underdiagnosed.

## Figures and Tables

**Table 1:** Mean pulmonary artery diameters (± standard deviation, SD) measured across all lung lobes in *Dirofilaria immitis*-seropositive symptomatic cats (Group A; n = 30) and seronegative asymptomatic cats (Group B; n = 8). Statistical significance: (*) *p* < 0.10; (**) *p* < 0.05.

Lung Lobe	Results	RESULTS OF THE PULMONARY ARTERY ACCORDING TO THE RESULTS OF ELISA TECHNIQUE				*p*-Value Mann–Whitney	Cohen’s d
		Valid N	Mean (mm)	SD	Median [Percentile 25 and 75] (mm)		
Left cranial (cranial subsegment)	Group A	30	1.19	0.40	1.20 [0.94–1.35]	0.082 *	0.242
	Group B	8	1.43	0.35	1.32 [1.17–1.69]		
Left cranial (caudal subsegment)	Group A	30	1.23	0.59	1.07 [0.85–1.55]	0.332	
	Group B	8	1.67	0.97	1.46 [0.94–2.19]		
Left caudal	Group A	30	2.14	0.60	2.26 [1.66–2.47]	0.221	
	Group B	8	2.44	0.36	2.33 [2.26–2.39]		
Right cranial	Group A	30	1.21	0.49	1.16 [0.86–1.38]	0.041 **	0.359
	Group B	8	1.57	0.49	1.43 [1.18–2.09]		
Right middle	Group A	30	1.02	0.49	0.86 [0.72–1.15]	0.332	
	Group B	8	1.16	0.59	0.97 [0.88–1.12]		
Right caudal	Group A	30	2.15	0.74	2.04 [1.75–2.59]	0.045 **	0.592
	Group B	8	2.74	0.71	2.78 [2.35–3.09]		
Accessory	Group A	30	0.97	0.22	0.92 [0.86–1.08]	0.538	
	Group B	8	1.16	0.49	1.03 [0.82–1.52]		

**Table 2:** Mean bronchial lumen diameters (± standard deviation: SD) measured across all lung lobes in *Dirofilaria immitis*-seropositive symptomatic cats (Group A; n = 30) and seronegative asymptomatic cats (Group B; n = 8). Abbreviations: IQR, interquartile range (25th–75th percentiles). Statistical significance: (**) *p* < 0.05.

Lung Lobe	Results	RESULTS OF THE BRONCHUS ACCORDING TO THE RESULTS OF THE ELISA TECHNIQUE				*p*-Value Mann–Whitney	Cohen’s d
		Valid N	Mean (mm)	SD	Median [IQR] (mm)		
Left cranial (cranial subsegment)	Group A	30	1.45	0.55	1.25 [1.11–1.71]	0.031 **	0.401
	Group B	8	1.05	0.31	0.91 [0.83–1.26]		
Left cranial (caudal subsegment)	Group A	30	1.37	0.51	1.21 [1.02–1.71]	0.314	
	Group B	8	1.27	0.75	1.02 [0.71–1.62]		
Left caudal	Group A	30	2.32	0.73	2.32 [1.80–2.75]	0.038 **	0.492
	Group B	8	1.82	0.28	1.81 [1.63–1.92]		
Right cranial	Group A	30	1.43	0.57	1.39 [0.98–1.72]	0.104	
	Group B	8	1.08	0.36	0.96 [0.80–1.32]		
Right middle	Group A	30	1.17	0.39	1.10 [0.91–1.41]	0.010 **	0.358
	Group B	8	0.82	0.35	0.71 [0.61–0.84]		
Right caudal	Group A	30	2.50	0.79	2.49 [1.90–2.93]	0.104	
	Group B	8	1.98	0.65	2.03 [1.46–2.54]		
Accessory	Group A	30	0.97	0.32	0.89 [0.75–1.15]	0.388	
	Group B	8	0.85	0.25	0.82 [0.70–0.97]		

**Table 3:** Pulmonary artery-to-vertebral body (A/VB) ratios for each lung lobe, comparing *Dirofilaria immitis*-seropositive symptomatic cats (Group A) and seronegative asymptomatic cats (Group B).

A/VB Ratio			Comparation of the A/VB Ratio for Each Lung Lobe Between Group A and B	
Lung Lobe	Group A (Median CI 95%)	Group B (Median CI 95%)	Group A vs. Group B (Median CI 95%)	Statistics and *p*-Value
Left cranial (cranial subsegment)	0.241 (0.209; 0.272)	0.274 (0.213; 0.335)	−0.033 (−0.102, 0.036)	t (0.980, 36) 0.334
Left cranial (caudal subsegment)	0.252 (0.2; 0.303)	0.317 (0.217; 0.417)	−0.065 (−0.177, 0.047)	t (1.173, 36) 0.248
Left caudal	0.433 (0.386; 0.48)	0.475 (0.384; 0.567)	−0.042 (−0.145, 0.060)	t (0.833, 36) 0.410
Right cranial	0.245 (0.205; 0.284)	0.301 (0.226; 0.377)	−0.057 (−0.142, 0.029)	t (1.348, 36) 0.186
Right middle	0.206 (0.167; 0.245)	0.221 (0.146; 0.296)	−0.015 (−0.100, 0.069)	t (0.364, 36) 0.718
Right caudal	0.433 (0.376; 0.491)	0.532 (0.421; 0.643)	−0.099 (−0.224, 0.026)	t (1.603, 36) 0.118
Accessory	0.196 (0.174; 0.218)	0.219 (0.176; 0.262)	−0.023 (−0.071, 0.026)	t (0.960, 36) 0.344

**Table 4:** Intragroup comparison of pulmonary artery-to-vertebral body (A/VB) ratios among lung lobes for *Dirofilaria immitis*-seropositive (Group A) and seronegative (Group B) cats. Statistical significance: (+) *p* < 0.10; (*) *p* < 0.05; (**) *p* < 0.01; (***) *p* < 0.001.

Pairs of Lung Lobes	Group A (Median CI 95%)	Group B (Median CI 95%)
Left cranial (cranial subsegment)–left caudal	−0.193 (−0.248, −0.137) ***	−0.201 (−0.309, −0.094) ***
Left cranial (cranial subsegment)–right caudal	−0.193 (−0.257, −0.128) ***	−0.258 (−0.383, −0.133) ***
Right cranial–left caudal	−0.189 (−0.249, −0.129) ***	−0.174 (−0.291, −0.058) **
Right cranial–right caudal	−0.189 (−0.257, −0.121) ***	−0.231 (−0.363, −0.099) **
Left cranial (caudal subsegment)–left caudal	−0.181 (−0.250, −0.113) ***	−0.159 (−0.291, −0.026) *
Left cranial (caudal subsegment)–right caudal	−0.181 (−0.257, −0.106) ***	−0.215 (−0.362, −0.069) **
Right middle–left caudal	−0.227 (−0.287, −0.168) ***	−0.254 (−0.370, −0.138) ***
Right middle–right caudal	−0.227 (−0.295, −0.159) ***	−0.311 (−0.443, −0.179) ***
Accessory–left caudal	−0.238 (−0.289, −0.186) ***	−0.257 (−0.356, −0.157) ***
Accessory–right caudal	−0.238 (−0.299, −0.177) ***	−0.313 (−0.431, −0.196) ***
Left cranial (cranial subsegment)–accessory	0.045 (0.007, 0.083) *	0.055 (−0.018, 0.129)
Right cranial–accessory	0.049 (0.005, 0.093) *	0.083 (−0.003, 0.169) +
Left cranial (caudal subsegment)–accessory	0.056 (0.001, 0.112) *	0.098 (−0.009, 0.206) +

**Table 5:** Bronchial lumen-to-vertebral body (B/VB) ratios for each lung lobe, comparing *Dirofilaria immitis*-seropositive symptomatic cats (Group A) and seronegative asymptomatic cats (Group B). Statistical significance: (+) *p* < 0.1; (*) *p* < 0.05.

B/VB Ratio			Comparation of the B/VB Ratio for Each Lung Lobe Between Group A and B	
Lung Lobe	Group A (Median CI 95%)	Group B (Median CI 95%)	Group A vs. Group B (Median CI 95%)	Statistics and *p*-Value
Left cranial (cranial subsegment)	0.289 (0.252; 0.327)	0.204 (0.132; 0.277)	0.085 (0.003, 0.167) *	t (2.100, 36) 0.043
Left cranial (caudal subsegment)	0.275 (0.234; 0.317)	0.242 (0.161; 0.323)	0.033 (−0.058, 0.124)	t (0.741, 36) 0.464
Left caudal	0.461 (0.41; 0.511)	0.358 (0.261; 0.456)	0.102 (−0.007, 0.212) +	t (1.891, 36) 0.067
Right cranial	0.286 (0.245; 0.326)	0.209 (0.130; 0.289)	0.076 (−0.013, 0.165) +	t (1.738, 36) 0.091
Right middle	0.233 (0.205; 0.260)	0.158 (0.104; 0.212)	0.075 (0.014, 0.135) *	t (2.487, 36) 0.018
Right caudal	0.498 (0.439; 0.556)	0.388 (0.275; 0.501)	0.110 (−0.017, 0.237) +	t (1.753, 36) 0.088
Accessory	0.193 (0.172; 0.214)	0.163 (0.123; 0.204)	0.029 (−0.016, 0.075)	t (1.304, 36) 0.200

**Table 6:** Intragroup comparison of bronchial lumen-to-vertebral body (B/VB) ratios among lung lobes for *Dirofilaria immitis*-seropositive (Group A) and seronegative (Group B) cats. Statistical significance: (+) *p* < 0.1; (*) *p* < 0.05; (**) *p* < 0.01; (***) *p* < 0.001.

Pairs of Lung Lobes	Group A (Median CI 95%)	Group B (Median CI 95%)
Left cranial (cranial subsegment)–left caudal	−0.171 (−0.233, −0.110) ***	−0.154 (−0.273, −0.034) *
Left cranial (cranial subsegment)–right caudal	−0.208 (−0.277, −0.140) ***	−0.183 (−0.316, −0.051) **
Right cranial–left caudal	−0.175 (−0.239, −0.111) ***	−0.149 (−0.272, −0.026) *
Right cranial–right caudal	−0.212 (−0.282, −0.142) ***	−0.178 (−0.314, −0.043) *
Left cranial (caudal subsegment)–left caudal	−0.185 (−0.250, −0.121) ***	−0.116 (−0.241, 0.008) +
Left cranial (caudal subsegment)–right caudal	−0.222 (−0.293, −0.152) ***	−0.146 (−0.282, −0.009) *
Right middle–left caudal	−0.228 (−0.285, −0.171) ***	−0.200 (−0.310, −0.091) **
Right middle–right caudal	−0.265 (−0.329, −0.201) ***	−0.230 (−0.353, −0.106) ***
Accessory–left caudal	−0.268 (−0.322, −0.214) ***	−0.195 (−0.299, −0.091) ***
Accessory–right caudal	−0.305 (−0.366, −0.243) ***	−0.224 (−0.343, −0.106) ***
Left cranial (cranial subsegment)–accessory	0.057 (0.011, 0.103) *	0.046 (−0.043, 0.135)
Right cranial–accessory	0.096 (0.054, 0.139) ***	0.041 (−0.041, 0.123)
Left cranial (caudal subsegment)–accessory	0.053 (0.005, 0.102) *	0.051 (−0.043, 0.145)

**Table 7:** Intra-operator repeatability analysis of CT-derived ratios in 10 randomly selected cats. Measurements were performed on five consecutive occasions per subject in the left and right caudal lobes. Abbreviations: SD: standard deviation (estimate of intra-operator error); Global mean: mean of all measurements (n = 25 per variable); CV: coefficient of variation; A/VB: pulmonary artery–vertebral body ratio; B/VB: bronchus–vertebral body ratio.

Lung Lobe	Measurement	SD	Global Mean	CV (%)
Left caudal	A/VB	0.0227	0.3546	6.40
	B/VB	0.0247	0.4959	4.98
Right caudal	A/VB	0.0157	0.3601	4.36
	B/VB	0.0232	0.5107	4.54

**Table 8:** Inter-observer reliability analysis between two independent researchers for A/VB and B/VB ratios in 10 randomly selected cats. Abbreviations: ICC, intraclass correlation coefficient (two-way random-effects model, single measurement, absolute agreement); SEM, standard error of measurement; Global mean, mean of all measurements (n = 10); CV, coefficient of variation calculated as SEM/global mean × 100; A/VB, pulmonary artery–vertebral body ratio; B/VB, bronchus–vertebral body ratio.

Lung Lobe	Measurement	ICC	SEM	Global Mean	CV (%)
Left caudal	A/VB	0.894	0.0177	0.3579	4.95%
	B/VB	0.859	0.0255	0.5002	5.10%
Right caudal	A/VB	0.978	0.0156	0.3551	4.39%
	B/VB	0.971	0.0266	0.5040	5.28%

## Data Availability

The original contributions presented in this study are included in the article. Further inquiries can be directed to the corresponding author.

## Abbreviations

The following abbreviations are used in this manuscript:

A/VB	Pulmonary artery/vertebral body ratio
B/VB	Bronchus/vertebral body ratio
CT	Computed tomography
HARD	Heartworm-Associated Respiratory Disease
PA	Pulmonary artery
T6	Sixth thoracic vertebra
